# Agility assessment using fuzzy logic approach: a case of healthcare dispensary

**DOI:** 10.1186/s12913-017-2332-y

**Published:** 2017-06-09

**Authors:** M. Suresh, Rojalin Patri

**Affiliations:** 1 Amrita School of Business, Amrita Vishwa Vidyapeetham, Amrita University, Coimbatore, 641 112 India; 2 Research Scholar, Amrita School of Business, Amrita Vishwa Vidyapeetham, Amrita University, Coimbatore, 641 112 India

**Keywords:** Healthcare agility, Dispensary operations, Agility assessment, Fuzzy agile index

## Abstract

**Background:**

Agile concepts are not only beneficial for manufacturing sector but also for service sector such as healthcare. However, assessment of agility has been predominantly done in manufacturing enterprises. This study demonstrates a means to measure agility of a healthcare organization by assessing agility of a university dispensary. Its contribution to the knowledge base is twofold. First, it proposes a means to measure the agility of a healthcare organization and second, it identifies the attributes that prevent agile performance and outlines the suggestive measure to enhance its agile capabilities.

**Method:**

A case study approach has been adopted and fuzzy logic has been employed to measure the agility of the case dispensary. At first, the measures of assessment which include four enablers, fifteen criteria and forty-five attributes have been identified from the literature and rated by the experts indicating the importance of the measures in the assessment. Then, the case dispensary has been assessed on those measures by collecting observed performance rating from decision makers. At last, Fuzzy logic has been applied on the performance rating data to analyze and interpret the agile capability of the dispensary.

**Results:**

The findings suggest that transparent information flow, adequate salary and bonuses for caregivers, reading error in medical descriptions, in house/nearby pathology laboratory services, technical up-gradation of dispensary equipments and facilities, minimization of patient throughput time and adequate training programme for safety practices are the attributes that weakens agile capability of the University dispensary. The current agility of the dispensary was found to be ‘Agile’ which is average in relation to the agility labels.

**Conclusion:**

Attributes such as transparent information flow, adequate salary and bonuses for caregivers, elimination of reading error in medical descriptions, in house/nearby pathology laboratory services, technical up-gradation of dispensary equipments and facilities, minimization of patient throughput time and adequate training programme for safety practices are extremely crucial for enhancing agile capability of a healthcare organization.

## Background

The term “Agility” came into existence in 1991 when a group of researchers at Iacocca Institute, Lehigh University conducted a study with 150 industry executives and referred the manufacturing practices observed during the study as agile practices [1]. Initially, agility was defined as using market knowledge and a virtual corporation to exploit profitable opportunities in a volatile market place [2]. Then it was referred as “the successful exploration of competitive bases (speed, flexibility, innovation pro-activity, quality and profitability) through the integration of reconfigurable resources and best practices in a knowledge-rich environment to provide customer- driven products and services in a fast changing market environment” [1]. Overtime, the definition went through further evolution and agility was defined as “the ability of an organization to respond rapidly to changes in demand both in terms of volume and variety” [3]. Another study translated agility to the power of moving quickly and having a quick resourceful and adaptable character [4]. Though the concept originated in manufacturing sector, it was found beneficial in service sectors as well; especially in healthcare sector [4]. Many studies and found that agile practices can help the healthcare organizations to meet the service demand which changes quickly and unpredictably and at the same time retain the competitive advantage over other players in the market [5, 6]. In addition, resilience and agility were found to be extremely crucial in healthcare [5]. Many studies in literature discussed how agility capabilities can help up-gradation of healthcare service quality [6]. Some studies also took an entirely different perspective by looking at agility as performance capability of an organization [7, 8]. As a result, the factors that drive agility of an organization have also been explored in the literature [9–11].

Acknowledging the importance of agility in healthcare, we continued our literature exploration in the context of how to assess agility of a healthcare organization. We found that assessment of agility has been predominantly done in manufacturing enterprises [12–17]. So far, agility of an organization has been assessed through several means such as index [13], system approach [12], graph theory [15], fuzzy data envelopment analysis [16, 17] and regression analysis [14]. Though a substantial amount of research endeavour has gone into assessment of agility in manufacturing enterprises, none of the studies has extended it to healthcare organizations. This leaves a gap in the existing literature as well as becomes the motivation behind this study. Here, we attempt to address the following research questions:

RQ1: How to measure agility of a healthcare organization?

RQ2: What are the attributes that influence agility of a healthcare organization?

RQ3: How to address those weak attributes to enhance agility?

Answer to these questions would help the researchers and managers in the field to identify the attributes that prevent agility of a healthcare organization and enforce suggestive measures to enhance its agile capabilities. Agile capability is referred as capability of an organization to prosper in a competitive environment and adapt quickly to the changing demands [18, 19].

## Methods

**Fig. 1:**
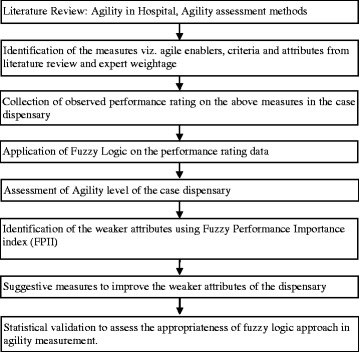
Framework to measure dispensary’ agility

In order to answer the research questions, we conducted a case analysis in the University dispensary located in India. The dispensary caters to 5000 students and 2000 staff members in the campus and as the university is actively involved in student exchange programs from various counties and visiting professors from different geographical locations, the dispensary aspires to enhance its service dynamics, responsiveness and efficiency to world standards. Therefore, this study considers the University dispensary as a case to assess and recommend suggestive measures to improve its agility. Fuzzy logic has been used to measure the current agility level of the dispensary and identify the attributes that pose a challenge to its agile performance. Fuzzy logic is preferred over other methods because it can take the linguistic data as input, analyze it and then express the results back in linguistic terms. Linguistic expressions are vague to interpret and have very small difference in meaning; for instance “Very bad” and “Worst”. Conversion of linguistic expressions into numerical values is difficult, and poses a challenge in terms of consistency and reliability. Fuzzy logic addresses these challenges by converting the linguistic variables into corresponding fuzzy intervals; also known as membership function, perform fuzzy operation and then convert it back into linguistic terms with the help of Fuzzy Agility Measure Index (FAMI). Apart from this, fuzzy logic also identifies the obstacles of the phenomenon. Many studies in literature have used fuzzy logic to assess several phenomena. One among them is agility assessment in manufacturing companies [20–22]. In this context, studies have developed an agility index [23] and adopted multi-grade fuzzy to measure agility level of the manufacturing organization [24]. Taking cues from these studies, this study adopts fuzzy logic to assess agility of a healthcare organization. Here, we use triangular fuzzy which assigns a three point interval or membership function to each of the linguistic variables. For example: the linguistic term “Worst” is captured in fuzzy interval (0, 0.5, 1.5). The assessment involves three major steps. First, a list of agile enablers, criteria and attributes that influence agile performance of a healthcare organization is identified. Second, the above measures (agile enablers, criteria and attributes) are assessed in the case hospital by collecting the observed performance rating from the decision makers. Third, fuzzy mathematical calculations are performed on the above performance rating data and agility of the healthcare organization is determined. The details regarding each of these steps are discussed below. Apart from this, a pictorial representation of the framework is presented in Fig 1.

### Step 1: identification of agile enablers, criteria and attributes

The first step, “identification of agile enablers, criteria and attributes” has been accomplished in this study by identifying a list of agile enablers, criteria and attributes from the literature, keeping the models for developed manufacturing organizations [1, 18, 20, 21, 25, 26] as reference. The list comprised of four enablers, fifteen criteria and forty-five attributes where each enabler branched out to a number of criteria and each criterion branched out to a set of attributes. The enablers, criteria and attributes put together constituted the measures of healthcare agility assessment and are presented in Table 1. Following the identification of these measures, five experts (E_1_, E_2,_…, E_5_) including three medical officers and two doctors from five different dispensaries in India were approached to capture the weightage of each enabler, criterion and attribute. The experts were asked to provide the weightage in terms of linguistic variables ranging from “Very low (VL)” to “Very High (VH)”. A sample question put forth to the experts to capture the weightage is given below.

**Table 1:** Conceptual model for dispensary agility assessment

Category of agile enablers	Agile criteria	Agile attributes
Agility through management driver *(AM*_*1*_ *)*	Management structure of dispensary *(AM*_*11*_ *)*	• Decentralised organisation structure *(AM*_*111*_ *)* • Team managed organisation *(AM*_*112*_ *)* • Transparent information flow *(AM*_*113*_ *)*
	Autonomy of work force *(AM*_*12*_ *)*	• Empowered work force *(AM*_*121*_ *)* • Autonomous decision making power *(AM*_*122*_ *)* • Responsibility of work *(AM*_*123*_ *)* • Work sharing culture *(AM*_*124*_ *)*
	Top management support *(AM*_*13*_ *)*	• Managing good relationship with caregivers *(AM*_*131*_ *)* • Adequate meeting with caregivers and work facilitation *(AM*_*132*_ *)* • Management involvement and transparent decisions *(AM*_*133*_ *)* • Adequate salary and bonuses for caregivers *(AM*_*134*_ *)*
Agility through technology driver *(AM*_*2*_ *)*	IT integration *(AM*_*21*_ *)*	• Patient information and medical history/records storages *(AM*_*211*_ *)* • Avoiding the medical descriptions reading error *(AM*_*212*_ *)* • Paperless work *(AM*_*213*_ *)*
	Automation of equipments *(AM*_*22*_ *)*	• The latest diagnostic equipment *(AM*_*221*_ *)* • Accuracy of diagnosis *(AM*_*222*_ *)* • Time saving through automation *(AM*_*223*_ *)*
	Change in service and technical process *(AM*_*23*_ *)*	• Up-gradation of pharmacy systems *(AM*_*231*_ *)* • Technical up-gradation of dispensary equipments and facilities *(AM*_*232*_ *)* • Change of patient pathway for minimising patient movements *(AM*_*233*_ *)*
	Patient flow and time management *(AM*_*24*_ *)*	• Patient flow monitoring and queuing system *(AM*_*241*_ *)* • Comfort system in the patient waiting place *(AM*_*242*_ *)* • Minimization of patient throughput time *(AM*_*243*_ *)*
	Visual Control *(AM*_*25*_ *)*	• Visual inspection of patient condition *(AM*_*251*_ *)* • Visually recognise the patient urgency *(AM*_*252*_ *)*
Agility through service strategy driver *(AM*_*3*_ *)*	Patients’ response adaptation *(AM*_*31*_ *)*	• Continuous quality improvement culture *(AM*_*311*_ *)* • Analyse the patients feedback and consider for future service improvements *(AM*_*312*_ *)*
	Status of the Caregivers *(AM*_*32*_ *)*	• Flexible caregivers to accept the advanced equipment implementation *(AM*_*321*_ *)* • Proper training about the new technology or systems *(AM*_*322*_ *)* • Multi-skilled and working on routine shifts *(AM*_*323*_ *)*
	Flexible work practices *(AM*_*33*_ *)*	• Manage the work responsibility within the available workforce *(AM*_*331*_ *)* • Mutual shift changes are allowed for caregivers *(AM*_*332*_ *)* • Flexible weekly offs of the caregivers with the prior information *(AM*_*333*_ *)* • Sharing the lessons learned from the day to day medical practices to their colleagues for better services *(AM*_*334*_ *)*
Agility through competitive driver *(AM*_*4*_ *)*	Patients’ service quality *(AM*_*41*_ *)*	• Ensuring the patients’ service quality *(AM*_*411*_ *)* • Progressive improvements of the patient recovery from sick *(AM*_*412*_ *)*
	Optimum treatment cost *(AM*_*42*_ *)*	• Maximum utilization of available equipment *(AM*_*421*_ *)* • Activity based cost classification system *(AM*_*422*_ *)* • Reduction of non-value added time and cost *(AM*_*423*_ *)*
	Patient and caregivers safety *(AM*_*43*_ *)*	• Adequate training programme for safety practices *(AM*_*431*_ *)* • Continuous maintenance of fire extinguishers in the dispensary *(AM*_*432*_ *)* • Maintain the safe and clean environment in-and-around the dispensary *(AM*_*433*_ *)*
	Caregivers involvement *(AM*_*44*_ *)*	• Creative thinking *(AM*_*441*_ *)* • Minimization of non-value added activities *(AM*_*442*_ *)* • Strong involvement and co-operations *(AM*_*443*_ *)*

Please mention how important is the following attribute for healthcare agility.

#### Decentralised organization structure

Very Low (  ) Low (  ) Fairly Low (  ) Medium (  ) Fairly High (  ) High (  ) Very High (  )

#### Team managed organization

Very Low (  ) Low (  ) Fairly Low (  ) Medium (  ) Fairly High (  ) High (  ) Very High (  )

For each of these linguistic variables from “Very low (VL)” to “Very High (VH)”, a corresponding fuzzy interval or membership function was assigned. For example: the linguistic variable “Very low (VL)” was assigned an interval (0, 0.05, 0.15) and “Very High (VH)” was assigned an interval (0.85, 0.95, 1.0). The intervals have been adopted from literature [20] and presented in Table 2. The linguistic weightage provided by the experts for enablers, criteria and attributes have been captured in Table 3, 4 and 5 respectively.

**Table 2:** Linguistic variables and fuzzy numbers for rating and weighting for agility

Performance Rating		Importance Weighting	
Linguistic variable	Fuzzy number	Linguistic variable	Fuzzy number
Worst (W)	(0,0.5,1.5)	Very Low (VL)	(0,0.05,0.15)
Very Poor (VP)	(1,2,3)	Low (L)	(0.1,0.2,0.3)
Poor (P)	(2,3.5,5)	Fairly Low (FL)	(0.2,0.35,0.5)
Fair (F)	(3,5,7)	Medium (M)	(0.3,0.5,0.7)
Good (G)	(5,6.5,8)	Fairly High (FH)	(0.5,0.65,0.8)
Very Good (VG)	(7,8,9)	High (H)	(0.7,0.8,0.9)
Excellent (E)	(8.5,9.5,10)	Very High (VH)	(0.85,0.95,1.0)

**Table 3:** Importance weight of agile capabilities of enablers

*AM*_*i*_	*N*_*i*_				
	*E*_*1*_	*E*_*2*_	*E*_*3*_	*E*_*4*_	*E*_*5*_
*AM*_*1*_	FH	M	M	FH	M
*AM*_*2*_	FH	M	FH	H	M
*AM*_*3*_	FH	H	H	H	H
*AM*_*4*_	H	H	H	VH	H

**Table 4:** Importance weight of agile capabilities of criteria

*AM*_*ij*_	*N*_*ij*_					*AM*_*ij*_	*N*_*ij*_				
	*E*_*1*_	*E*_*2*_	*E*_*3*_	*E*_*4*_	*E*_*5*_		*E*_*1*_	*E*_*2*_	*E*_*3*_	*E*_*4*_	*E*_*5*_
*AM*_*11*_	H	FH	H	H	FH	*AM*_*31*_	M	FH	FH	H	VH
*AM*_*12*_	H	H	H	H	H	*AM*_*32*_	FH	FH	FH	H	FH
*AM*_*13*_	VH	H	H	H	H	*AM*_*33*_	H	H	H	H	VH
*AM*_*21*_	FH	FH	FH	H	FH	*AM*_*41*_	VH	VH	VH	H	VH
*AM*_*22*_	FH	H	H	H	FH	*AM*_*42*_	FH	M	M	FH	FH
*AM*_*23*_	H	H	H	FH	H	*AM*_*43*_	H	VH	H	VH	VH
*AM*_*24*_	H	H	VH	VH	H	*AM*_*44*_	FH	FH	H	H	FH
*AM*_*25*_	M	FH	M	FH	H

**Table 5:** Importance weight and performance rating of agile capabilities of attributes

AM_ijk_	*N*_*ijk*_					*O*_*ijk*_				
	*E*_*1*_	*E*_*2*_	*E*_*3*_	*E*_*4*_	*E*_*5*_	*D*_*1*_	*D*_*2*_	*D*_*3*_	*D*_*4*_	*D*_*5*_
AM_111_	H	FH	M	FH	FH	F	F	P	P	F
AM_112_	M	FH	M	FH	H	F	F	G	F	F
AM_113_	H	VH	H	H	VH	F	G	F	P	F
AM_121_	FH	FH	FH	H	FH	F	G	F	P	F
AM_122_	H	FH	H	FH	FH	F	F	P	F	G
AM_123_	H	FH	FH	FH	H	G	G	G	VG	VG
AM_124_	H	FH	H	FH	FH	F	F	F	G	G
AM_131_	FH	FH	H	H	H	G	F	F	G	G
AM_132_	H	FH	FH	FH	FH	F	F	P	F	F
AM_133_	H	H	FH	H	H	F	F	F	F	G
AM_134_	H	VH	H	H	VH	F	F	F	F	G
AM_211_	FH	M	FH	M	M	G	F	G	G	G
AM_212_	H	H	H	VH	H	G	G	G	G	G
AM_213_	FH	FL	M	FL	FL	P	VP	VP	P	VP
AM_221_	FH	M	M	FH	FH	P	P	P	F	F
AM_222_	H	H	H	H	H	F	F	G	G	G
AM_223_	FH	FH	M	H	FH	P	P	VP	VP	VP
AM_231_	FH	H	H	H	FH	F	F	F	F	G
AM_232_	FH	FH	FH	H	H	P	P	P	P	F
AM_233_	FH	M	M	M	FH	G	G	VG	G	VG
AM_241_	M	M	FH	M	FH	F	G	F	G	G
AM_242_	M	FH	M	M	M	G	G	F	G	G
AM_243_	H	H	H	H	H	F	F	P	F	F
AM_251_	H	FH	H	H	FH	G	G	G	G	G
AM_252_	H	H	H	H	H	G	G	G	G	VG
AM_311_	FH	FH	H	FH	FH	F	G	F	G	VG
AM_312_	FH	M	M	M	M	F	F	F	F	G
AM_321_	M	FH	M	FH	M	G	G	G	G	VG
AM_322_	FH	H	H	FH	H	F	F	F	F	F
AM_323_	FH	M	M	M	FH	G	G	VG	VG	VG
AM_331_	FH	H	H	H	FH	G	G	G	G	VG
AM_332_	M	M	M	M	FH	G	G	G	G	G
AM_333_	FH	M	M	M	FH	G	G	G	G	G
AM_334_	FH	M	FH	M	FH	G	F	F	G	G
AM_411_	H	H	FH	H	H	G	G	G	G	VG
AM_412_	H	FH	FH	FH	H	G	G	G	G	G
AM_421_	M	FH	M	M	FH	G	VG	G	VG	VG
AM_422_	FH	M	M	M	FH	G	G	G	VG	VG
AM_423_	FH	FH	H	FH	H	F	F	F	F	G
AM_431_	FH	H	H	H	H	F	F	F	F	F
AM_432_	FH	M	FH	FH	FH	VG	VG	VG	E	E
AM_433_	FH	FH	FH	H	H	G	G	VG	G	VG
AM_441_	FH	FH	FH	H	FH	G	G	G	G	VG
AM_442_	FH	FH	H	H	H	F	F	F	F	G
AM_443_	FH	FH	FH	FH	FH	F	G	F	F	VG

### Step 2: collection of observed performance rating on the measures

In the second step, a questionnaire was shared among five decision makers (D_1_, D_2_,…, D_5_) who are doctors in the case dispensary to provide an observed performance rating indicating where the dispensary stands in terms of each attribute. Here, the performance rating is collected for attributes alone because during fuzzy analysis, the attribute ratings are aggregated to criteria rating and criteria rating to enablers rating. The computations pertaining to this aggregation have been discussed in the sub section, “Details of fuzzy calculation steps”. The attribute rating was captured in linguistic variables ranging from “Worst (W)” to “Excellent (E)”. A sample question put forth to the decision makers is given below.

Please rate the dispensary indicating where it stands with respect to the attributes underlined below:

#### Decentralised organization structure

Worst (  ) Very poor (  ) Poor (  ) Fair (  ) Good (  ) Very Good (  ) Excellent (  )

#### Team managed organization

Worst (  ) Very poor (  ) Poor (  ) Fair (  ) Good (  ) Very Good (  ) Excellent (  )

For each of these linguistic variables from “Worst (W)” to “Excellent (E)”, a corresponding fuzzy interval or membership function was assigned. For example: the linguistic variable “Worst (W)” was assigned an interval (0, 0.5, 1.5) and “Excellent (E)” was assigned an interval (8.5, 9.5, 10). The intervals have been adopted from literature [20] and presented in Table 2. The performance rating provided by the decision makers is shown in Table 5.

### Step 3: application of fuzzy calculations on the observed performance rating

In this step, fuzzy calculations are performed on the performance rating data collected in step 2. The details regarding the calculation are discussed in sub section “Details of fuzzy calculation steps” and the notations used are presented in Table 6.

**Table 6:** Notations used for fuzzy logic agility assessment model

*Indices*	*Abbreviations*
*O*_*i*_	fuzzy performance rating for agility of *i* ^th^ enabler
*O*_*ij*_	fuzzy performance rating for agility of *j* ^th^ criterion in *i* ^th^ enabler
*O*_*ijk*_	fuzzy observed rating for agility of *k* ^*th*^ attribute of *j* ^*th*^ criterion of *i* ^*th*^ enabler
*N*_*i*_	fuzzy importance weight for agility of *i* ^*th*^ enabler
*N*_*ij*_	fuzzy importance weight for agility of *j* ^*th*^ criterion in *i* ^*th*^ enabler
*N*_*ijk*_	fuzzy importance weight for agility of *k* ^*th*^ attribute of *j* ^*th*^ criterion in *i* ^*th*^ enabler
*AM*_*i*_	agility measure of *i* ^*th*^ enabler
*AM*_*ij*_	agility measure of *j* ^*th*^ criterion in *i* ^*th*^ enabler
*AM*_*ijk*_	agility measure of *k* ^*th*^ attribute of *j* ^*th*^ criterion in *i* ^*th*^ enabler
*FAMI*	fuzzy agility measure index
*AL*_*i*_	fuzzy number of agile level for natural-language expression
*fFAMI (x)*	triangular fuzzy number of *FAMI*
*fAL*_*i*_ *(x)*	triangular fuzzy number of *AL*_*i*_

#### Details of fuzzy calculation steps

At first, the fuzzy intervals assigned to the expert weightage in step 1 and performance ratings in step 2 are aggregated using average operation method. The decision for adopting this method has been taken following the literature [27]. An instance of ‘average fuzzy weightage’ and ‘average fuzzy performance rating’ calculation for the attribute “Decentralised organisation structure” (AM_111_) is shown below.

$$ \begin{array}{l}\mathrm{Formula}\ \mathrm{of}\ \mathrm{average}\ \mathrm{operation}\ \mathrm{method} = \left({\mathrm{a}}_1{\mathrm{b}}_1{\mathrm{c}}_1\right) +, \dots, + \left({\mathrm{a}}_{\mathrm{n}}{\mathrm{b}}_{\mathrm{n}}{\mathrm{c}}_{\mathrm{n}}\right) = \\ {}\left[\left({\mathrm{a}}_1+\dots + {\mathrm{a}}_{\mathrm{n}}\right)/\mathrm{n},\ \left({\mathrm{b}}_1+\dots + {\mathrm{b}}_{\mathrm{n}}\right)/\mathrm{n},\ \left({\mathrm{c}}_1+\dots + {\mathrm{c}}_{\mathrm{n}}\right),/,\mathrm{n}\right]\end{array} $$

$$ \begin{array}{l}\mathrm{Average}\ \mathrm{fuzzy}\ \mathrm{weight}\ \mathrm{of}\ \mathrm{the}\ \mathrm{attribute} = \left[\mathrm{H}+\mathrm{FH}+\mathrm{M}+\mathrm{FH}+\mathrm{FH}\right]/5\ \\ {} = \left(0.7\ 0.8\ 0.9\right)/5,\ \left(0.5\ 0.65\ 0.8\right)/5,\ \left(0.3\ 0.5\ 0.7\right)\ /5,\left(0.5\ 0.65\ 0.8\right)\ /5,\left(0.5\ 0.65\ 0.8\right)\Big]/5 = \left(0.5\ 0.65\ 0.8\right)\end{array} $$

$$ \begin{array}{l}\mathrm{Average}\ \mathrm{performance}\ \mathrm{rating}\ \mathrm{of}\ \mathrm{the}\ \mathrm{attribute} = \left[\mathrm{F}+\mathrm{F}+\mathrm{P}+\mathrm{P}+\mathrm{F}\right]/5\ \\ {} = \left[\left(3\ 5\ 7\right)\ /5,\ \left(3\ 5\ 7\right)\ /5,\ \left(2\ 3.5\ 5\right)\ /5,\ \left(2\ 3.5\ 5\right)\ /5,\ \left(3\ 5\ 7\right)\right]/5 = \left(2.6\ 4.4\ 6.2\right)\end{array} $$

In the next step, the aggregate performance ratings of the attributes is translated into criteria rating and the criteria rating is translated into enabler rating using equation 1 and 2 respectively. Table 7 captures the criteria ratings and Table 8 captures the enabler ratings of the study. An instance of criteria rating calculation for “management structure of dispensary” *O*_*11*_ *(AM*_*11*_ *)* and enabler rating calculation for “agility through management drivers” *(AM*_*1*_ *)* are demonstrated below.

**Table 7:** Fuzzy index of agile capabilities of criteria rating

Agile Criteria	Agile Attributes	Fuzzy performance average rating *(≈O*_*ijk*_ *)*	Attributes importance average weight *(≈N*_*ijk*_ *)*	Criteria rating
*AM*_*11*_	AM_111_	(2.6 4.4 6.2)	(0.5 0.65 0.8)	(3.08 4.90 6.73)
	AM_112_	(3.4 5.3 7.2)	(0.46 0.62 0.78)	
	AM_113_	(3.2 5 6.8)	(0.76 0.86 0.94)	
AM_12_	AM_121_	(3.2 5 6.8)	(0.54 0.68 0.82)	(4.01 5.68 7.35)
	AM_122_	(3.2 5 6.8)	(0.58 0.71 0.84)	
	AM_123_	(5.8 7.1 8.4)	(0.58 0.71 0.84)	
	AM_124_	(3.8 5.6 7.4)	(0.58 0.71 0.84)	
AM_13_	AM_131_	(4.2 5.9 7.6)	(0.62 0.74 0.86)	(3.46 5.31 7.15)
	AM_132_	(2.8 4.7 6.6)	(0.54 0.68 0.82)	
	AM_133_	(3.4 5.3 7.2)	(0.66 0.77 0.88)	
	AM_134_	(3.4 5.3 7.2)	(0.76 0.86 0.94)	
AM_21_	AM_211_	(4.6 6.2 7.8)	(0.38 0.56 0.74)	(4.16 5.47 6.82)
	AM_212_	(5 6.5 8)	(0.73 0.83 0.92)	
	AM_213_	(1.4 2.6 3.8)	(0.28 0.44 0.6)	
AM_22_	AM_221_	(2.4 4.1 5.8)	(0.42 0.59 0.76)	(2.87 4.33 5.80)
	AM_222_	(4.2 5.9 7.6)	(0.7 0.8 0.9)	
	AM_223_	(1.4 2.6 3.8)	(0.5 0.65 0.8)	
AM_23_	AM_231_	(3.4 5.3 7.2)	(0.62 0.74 0.86)	(3.54 5.27 6.94)
	AM_232_	(2.2 3.8 5.4)	(0.58 0.71 0.84)	
	AM_233_	(5.8 7.1 8.4)	(0.38 0.56 0.74)	
AM_24_	AM_241_	(4.2 5.9 7.6)	(0.38 0.56 0.74)	(3.60 5.47 7.28)
	AM_242_	(4.6 6.2 7.8)	(0.34 0.53 0.72)	
	AM_243_	(2.8 4.7 6.6)	(0.7 0.8 0.9)	
AM_25_	AM_251_	(5 6.5 8)	(0.62 0.74 0.86)	(5.21 6.65 8.10)
	AM_252_	(5.4 6.8 8.2)	(0.7 0.8 0.9)	
AM_31_	AM_311_	(4.6 6.2 7.8)	(0.54 0.68 0.82)	(4.14 5.80 7.52)
	AM_312_	(3.4 5.3 7.2)	(0.34 0.53 0.72)	
AM_32_	AM_321_	(5.4 6.8 8.2)	(0.38 0.56 0.74)	(4.54 6.26 7.88)
	AM_322_	(3 5 7)	(0.62 0.74 0.86)	
	AM_323_	(6.2 7.4 8.6)	(0.38 0.56 0.74)	
AM_33_	AM_331_	(5.4 6.8 8.2)	(0.62 0.74 0.86)	(4.95 6.44 7.96)
	AM_332_	(5 6.5 8)	(0.34 0.53 0.72)	
	AM_333_	(5 6.5 8)	(0.38 0.56 0.74)	
	AM_334_	(4.2 5.9 7.6)	(0.42 0.59 0.76)	
AM_41_	AM_411_	(5.4 6.8 8.2)	(0.66 0.77 0.88)	(5.21 6.65 8.10)
	AM_412_	(5 6.5 8)	(0.58 0.71 0.84)	
AM_42_	AM_421_	(6.2 7.4 8.6)	(0.38 0.56 0.74)	(4.87 6.49 8.03)
	AM_422_	(5.8 7.1 8.4)	(0.38 0.56 0.74)	
	AM_423_	(3.4 5.3 7.2)	(0.58 0.71 0.84)	
AM_43_	AM_431_	(3 5 7)	(0.66 0.77 0.88)	(5.2 6.77 8.22)
	AM_432_	(7.6 8.6 9.4)	(0.46 0.62 0.78)	
	AM_433_	(5.8 7.1 8.4)	(0.58 0.71 0.84)	
AM_44_	AM_441_	(5.4 6.8 8.2)	(0.54 0.68 0.82)	(4.29 5.98 7.66)
	AM_442_	(3.4 5.3 7.2)	(0.62 0.74 0.86)	
	AM_443_	(4.2 5.9 7.6)	(0.5 0.65 0.8)

**Table 8:** Fuzzy index of agile capabilities of enablers rating and FAMI

Agile Enabler	Agile Criteria	Criteria rating *(O*_*ij*_ *)*	Criteria importance average weight *(≈N*_*ij*_ *)*	Enabler rating *(O*_*i*_ *)*	Enabler importance average weight *(≈N*_*i*_ *)*	Fuzzy Agile Measure Index
*AM*_*1*_	*AM*_*11*_	(3.08 4.90 6.73)	(0.62 0.74 0.86)	(3.54 5.31 7.09)	(0.38 0.56 0.74)	(4.36 5.93 7.50)
	AM_12_	(4.01 5.68 7.35)	(0.7 0.8 0.9)			
	AM_13_	(3.46 5.31 7.15)	(0.73 0.83 0.92)			
AM_2_	AM_21_	(4.16 5.47 6.82)	(0.54 0.68 0.82)	(3.78 5.49 6.97)	(0.46 0.62 0.78)	
	AM_22_	(2.87 4.33 5.80)	(0.62 0.74 0.86)			
	AM_23_	(3.54 5.27 6.94)	(0.66 0.77 0.88)			
	AM_24_	(3.60 5.47 7.28)	(0.76 0.86 0.94)			
	AM_25_	(5.21 6.65 8.10)	(0.46 0.62 0.78)			
AM_3_	AM_31_	(4.14 5.80 7.52)	(0.57 0.71 0.84)	(4.58 6.18 7.79)	(0.66 0.77 0.88)	
	AM_32_	(4.54 6.26 7.88)	(0.54 0.68 0.82)			
	AM_33_	(4.95 6.44 7.96)	(0.73 0.83 0.92)			
AM_4_	AM_41_	(5.21 6.65 8.10)	(0.82 0.92 0.98)	(4.95 6.50 8.01)	(0.73 0.83 0.92)	
	AM_42_	(4.87 6.49 8.03)	(0.42 0.59 0.76)			
	AM_43_	(5.2 6.77 8.22)	(0.79 0.89 0.96)			
	AM_44_	(4.29 5.98 7.66)	(0.58 0.71 0.84)			

1 $$ {O}_{ij}=\frac{{\displaystyle \sum_{k=1}^K\left({O}_{ij k}\otimes {N}_{ij k}\right)}}{{\displaystyle \sum_{k=1}^K{O}_{ij k}}} $$

2 $$ {O}_i=\frac{{\displaystyle \sum_{j=1}^J\left({O}_{i j}\otimes {N}_{i j}\right)}}{{\displaystyle \sum_{j=1}^J{O}_{i j}}} $$

$$ {O}_{11}\left( A{M}_{11}\right)=\frac{\left[\left(5.8\ 7.1\kern0.5em 8.4\right) \otimes \left(0.54\kern0.5em 0.68\kern0.5em 0.82\right)\oplus \left(5.4\kern0.5em 6.8\kern0.5em 8.2\right)\otimes \left(0.32\kern0.5em 0.5\kern0.5em 0.68\right)\oplus \left(3.8\kern0.5em 5.6\ 7.4\right)\otimes \left(0.24\kern0.5em 0.41\ 0.58\right)\right]}{\left[\left(0.54\kern0.5em 0.68\kern0.5em 0.82\right)\oplus \left(0.32\kern0.5em 0.5\kern0.75em 0.68\right)\oplus \left(0.24\kern0.5em 0.41\kern0.5em 0.58\right)\right]} $$

$$ {O}_{11}\left( A{M}_{11}\right) = \left(5.25\ 6.62\ 8.05\right) $$

$$ {\mathrm{O}}_1\left( A{M}_1\right)=\frac{\left[\left(2.6\kern0.5em 4.4\kern0.5em 6.2\right)\otimes \left(0.5\kern0.75em 0.65\kern0.75em 0.8\right)\oplus \left(3.4\kern0.75em 5.3\kern0.75em 7.2\right)\otimes \left(0.46\kern0.75em 0.62\kern0.75em 0.78\right)\oplus \left(3.2\kern0.75em 5\kern0.75em 6.8\right)\otimes \left(0.76\kern0.75em 0.86\kern0.75em 0.94\right)\right]}{\left[\left(0.5\kern0.75em 0.65\kern0.75em 0.8\right)\oplus \left(0.46\kern0.75em 0.62\kern0.75em 0.78\right)\oplus \left(0.76\kern0.75em 0.86\kern0.75em 0.94\right)\right]} $$

$$ {O}_1\left( A{M}_1\right) = \left(3.08\ 4.90\ 6.73\right) $$

After obtaining the ratings for criteria and enablers, the Fuzzy Agility Measure Index (FAMI) of the dispensary is calculated using equation 3. FAMI represents the overall agility of the dispensary and the calculation is shown below.

3 $$ FAMI=\frac{{\displaystyle \sum_{i=1}^I\left({O}_i\otimes {N}_i\right)}}{{\displaystyle \sum_{i=1}^I{O}_i}} $$

$$ FAMI=\frac{\left[\begin{array}{l}\left(3.54\kern0.5em 5.31\kern0.5em 7.09\right) \otimes \left(0.38\kern0.5em 0.56\kern0.5em 0.74\right)\oplus \left(3.78\kern0.5em 5.49\kern0.5em 6.97\right)\otimes \left(0.46\kern0.5em 0.62\kern0.5em 0.78\right)\oplus \\ {}\left(4.58\kern0.5em 6.18\kern0.5em 7.79\right)\otimes \left(0.66\kern0.5em 0.77\kern0.5em 0.88\right)\oplus \left(4.95\kern0.5em 6.50\kern0.5em 8.01\right)\otimes \left(0.73\kern0.5em 0.83\kern0.5em 0.92\right)\end{array}\right]}{\left[\left(0.38\kern0.5em 0.56\kern0.5em 0.74\right)\oplus \left(0.46\kern0.5em 0.62\kern0.5em 0.78\right)\oplus \left(0.66\kern0.5em 0.77\kern0.5em 0.88\right)\oplus \left(0.73\kern0.5em 0.83\kern0.5em 0.92\right)\right]}=\left(4.36\kern0.5em 5.93\kern0.5em 7.50\right) $$

#### Euclidean distance method

Upon obtaining the FAMI, it is then converted back into linguistic terms using Euclidean distance method. Euclidean distance method is considered as the most intuitive method for humans to calculate perceived proximity [20, 22, 27]. In this method, five linguistic terms known as “natural language expression set of Agility Label (AL)” are adopted from literature [20] and for each agility label, the Euclidean distance (*D)* is calculated following equation 4. The minimum distance or *D* value is considered as the agility label of the organization in linguistic terms. Table 9 captures the linguistic agility labels and the corresponding fuzzy intervals. The calculation of Euclidean distance is shown below.

**Table 9:** Agility factor and fuzzy determination

Natural Language expression set of Agility Label (AL)	Fuzzy determination
Extremely Agile (EA)	(7, 8.5, 10)
Very Agile (VA)	(5.5, 7, 8.5)
Agile (A)	(3.5 5 6.5)
Fairly Agile (FA)	(1.5, 3, 4.5)
Slowly Agile (SA)	(0, 1.5, 3)

4 $$ D\left( FAMI, A{L}_i\right)=\sqrt{{\displaystyle \sum_x{\displaystyle {\left( fFAMI(x)- fA{L}_i(x)\right)}^2}}} $$

$$ \mathrm{FAMI}\ \mathrm{f}\mathrm{o}\mathrm{r}\ \mathrm{the}\ \mathrm{case}\ \mathrm{dispensary}:\ \left(4.36\ 5.93\ 7.50\right) $$

$$ \mathrm{D}\ \left(\mathrm{FAMI},\ \mathrm{EA}\right) = \kern0.5em {\left\{{\left(4.36\hbox{-} 7\right)}^2\kern0.5em +\kern0.5em {\left(5.93\hbox{-} 8.5\right)}^2\kern0.5em +\kern0.5em {\left(7.50\hbox{-} 10\right)}^2\right\}}^{1/2} = 4.45 $$

$$ \mathrm{D}\ \left(\mathrm{FAMI},\ \mathrm{VA}\right) = \kern0.75em {\left\{{\left(4.36-5.5\right)}^2\kern0.5em +\kern0.5em {\left(5.93-7\right)}^2\kern0.5em +\kern0.5em {\left(7.50-8.5\right)}^2\right\}}^{1/2} = 1.86 $$

$$ \mathrm{D}\left(\mathrm{FAMI},\mathrm{A}\right)=\kern0.75em {\left\{{\left(4.36-3.5\right)}^2\kern0.5em +\kern0.5em {\left(5.93-5.0\right)}^2+\kern0.5em {\left(7.50-6.5\right)}^2\right\}}^{1/2}=\mathbf{1.61} $$

$$ \mathrm{D}\ \left(\mathrm{FAMI},\ \mathrm{FA}\right)=\kern0.75em {\left\{{\left(4.36-1.5\right)}^2\kern0.5em +\kern0.5em {\left(5.93-3\right)}^2+{\left(7.50-4.5\right)}^2\right\}}^{1/2}=5.07 $$

$$ \mathrm{D}\ \left(\mathrm{FAMI},\ \mathrm{SA}\right)=\kern0.75em {\left\{{\left(4.36-0\right)}^2\kern0.5em +\kern0.5em {\left(5.93-1.5\right)}^2+{\left(7.50-3\right)}^2\right\}}^{1/2}=7.67 $$

**Fig. 2:**
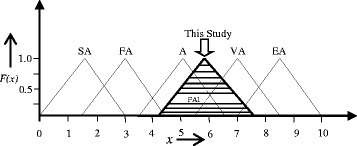
Linguistic levels to match fuzzy-agility-index

As the *D* value is minimum for the label “Agile”, the AL of the case dispensary was considered to be “Agile” in linguistic terms. Fig. 2 represents the agility label of the dispensary pictorially.

#### Fuzzy performance importance index (FPII)

Fuzzy Performance Importance Index (FPII) calculation is performed to identify the attributes that pose a challenge to the phenomenon which in this study is agile capability. The FPII computation consists of two steps: first is the calculation of FPII following equation 5 and second is the development of a ranking score for each attribute following centroid method shown in equation 6. An instance of FPII and ranking score calculation for attribute “Decentralised organisation structure (AM_111_)” is shown below following the equations.

5 $$ F P I{I}_{ijk}={W\kern-0.3em }_{ijk}\times {O}_{ijk}; $$

where, $$ {W}_{ijk}\kern0.5em =\kern0.5em \left(1,\kern0.5em 1,\kern0.5em 1\right)\kern0.5em -\kern0.5em {N}_{ijk} $$

6 $$ \left(\mathrm{a} + 4\mathrm{b} + \mathrm{c}\right)/6 $$

$$ {W}_{111}=\left(1,\ 1,\ 1\right)-\left(0.5\ 0.65\ 0.8\right)\to \left(0.5\ 0.35\ 0.2\right) $$

$$ F P I{I}_{111}=\left(0.5\ 0.35\ 0.2\right)\times \left(2.6\ 4.4\ 6.2\right)\to \left(1.3\ 1.54\ 1.24\right) $$

$$ \mathrm{Ranking}\ \mathrm{score}\ \mathrm{of}\ \mathrm{A}{\mathrm{M}}_{111}=\left[\left(1.3+\left(4\times 1.54\right)+1.24\right)\right)/6\Big]\to 1.45 $$

After obtaining the ranking scores, the dispensary management was consulted to decide the threshold. The reason behind consulting the management to set the threshold is that the management has to take a decision on how high the agile capability of the dispensary will be raised going further. If the management is not ready to aim high, then it is more likely that they would choose a lower threshold whereas it reverses if the management is fully in for it. In this study, after management consultation, ‘1.1’ was set as the threshold and the attributes below 1.1 was identified as weak attributes. As a result, seven attributes viz. transparent information flow, adequate salary and bonuses for caregivers, medical descriptions reading error, in house/nearby pathology laboratory services, technical up-gradation of dispensary equipments and facilities, minimization of patient throughput time and adequate training programme for safety practices were found to be the critical or weak for the university dispensary. Table 10 captures the FPII and ranking score of the attributes and Table 11 presents the suggestive measures for the critical attributes.

**Table 10:** FPII of 45 agile capabilities of current level of dispensary

Agile Attributes	Fuzzy performance average rating	*W*_*ijk*_ *= (1, 1, 1) − N*_*ijk*_	Fuzzy performance importance index	Ranking score
AM_111_	(2.6 4.4 6.2)	(0.5 0.35 0.2)	(1.3 1.54 1.24)	1.45
AM_112_	(3.4 5.3 7.2)	(0.54 0.38 0.22)	(1.84 2.01 1.58)	1.91
AM_113_	(3.2 5 6.8)	(0.24 0.14 0.06)	(0.77 0.7 0.41)	0.66^a^
AM_121_	(3.2 5 6.8)	(0.46 0.32 0.18)	(1.47 1.6 1.22)	1.52
AM_122_	(3.2 5 6.8)	(0.42 0.29 0.16)	(1.34 1.45 1.09)	1.37
AM_123_	(5.8 7.1 8.4)	(0.42 0.29 0.16)	(2.44 2.06 1.34)	2.00
AM_124_	(3.8 5.6 7.4)	(0.42 0.29 0.16)	(1.60 1.62 1.18)	1.55
AM_131_	(4.2 5.9 7.6)	(0.38 0.26 0.14)	(1.60 1.53 1.06)	1.47
AM_132_	(2.8 4.7 6.6)	(0.46 0.32 0.18)	(1.28 1.50 1.19)	1.41
AM_133_	(3.4 5.3 7.2)	(0.34 0.23 0.12)	(1.16 1.22 0.86)	1.15
AM_134_	(3.4 5.3 7.2)	(0.24 0.14 0.06)	(0.82 0.74 0.43)	0.70^a^
AM_211_	(4.6 6.2 7.8)	(0.62 0.44 0.26)	(2.85 2.73 2.03)	2.63
AM_212_	(5 6.5 8)	(0.27 0.17 0.08)	(1.35 1.10 0.64)	1.07^a^
AM_213_	(1.4 2.6 3.8)	(0.72 0.56 0.4)	(1.01 1.46 1.52)	1.39
AM_221_	(2.4 4.1 5.8)	(0.58 0.41 0.24)	(1.39 1.68 1.39)	1.58
AM_222_	(4.2 5.9 7.6)	(0.3 0.2 0.1)	(1.26 1.18 0.76)	1.12
AM_223_	(1.4 2.6 3.8)	(0.5 0.35 0.2)	(0.7 0.91 0.76)	0.85^a^
AM_231_	(3.4 5.3 7.2)	(0.38 0.26 0.14)	(1.29 1.38 1.01)	1.30
AM_232_	(2.2 3.8 5.4)	(0.42 0.29 0.16)	(0.92 1.10 0.86)	1.03^a^
AM_233_	(5.8 7.1 8.4)	(0.62 0.44 0.26)	(3.6 3.12 2.18)	3.05
AM_241_	(4.2 5.9 7.6)	(0.62 0.44 0.26)	(2.60 2.59 1.98)	2.49
AM_242_	(4.6 6.2 7.8)	(0.66 0.47 0.28)	(3.04 2.91 2.18)	2.81
AM_243_	(2.8 4.7 6.6)	(0.3 0.2 0.1)	(0.84 0.94 0.66)	0.87^a^
AM_251_	(5 6.5 8)	(0.38 0.26 0.14)	(1.9 1.69 1.12)	1.63
AM_252_	(5.4 6.8 8.2)	(0.3 0.2 0.1)	(1.62 1.36 0.82)	1.31
AM_311_	(4.6 6.2 7.8)	(0.46 0.32 0.18)	(2.12 1.98 1.40)	1.91
AM_312_	(3.4 5.3 7.2)	(0.66 0.47 0.28)	(2.24 2.49 2.02)	2.37
AM_321_	(5.4 6.8 8.2)	(0.62 0.44 0.26)	(3.35 2.99 2.13)	2.91
AM_322_	(3 5 7)	(0.38 0.26 0.14)	(1.14 1.3 0.98)	1.22
AM_323_	(6.2 7.4 8.6)	(0.62 0.44 0.26)	(3.84 3.26 2.24)	3.18
AM_331_	(5.4 6.8 8.2)	(0.38 0.26 0.14)	(2.05 1.77 1.15)	1.71
AM_332_	(5 6.5 8)	(0.66 0.47 0.28)	(3.3 3.06 2.24)	2.96
AM_333_	(5 6.5 8)	(0.62 0.44 0.26)	(3.1 2.86 2.08)	2.77
AM_334_	(4.2 5.9 7.6)	(0.58 0.41 0.24)	(2.44 2.42 1.82)	2.32
AM_411_	(5.4 6.8 8.2)	(0.34 0.23 0.12)	(1.84 1.56 0.98)	1.51
AM_412_	(5 6.5 8)	(0.42 0.29 0.16)	(2.1 1.88 1.28)	1.82
AM_421_	(6.2 7.4 8.6)	(0.62 0.44 0.26)	(3.84 3.26 2.24)	3.18
AM_422_	(5.8 7.1 8.4)	(0.62 0.44 0.26)	(3.60 3.12 2.18)	3.05
AM_423_	(3.4 5.3 7.2)	(0.42 0.29 0.16)	(1.43 1.54 1.15)	1.45
AM_431_	(3 5 7)	(0.34 0.23 0.12)	(1.02 1.15 0.84)	1.08^a^
AM_432_	(7.6 8.6 9.4)	(0.54 0.38 0.22)	(4.10 3.27 2.07)	3.21
AM_433_	(5.8 7.1 8.4)	(0.42 0.29 0.16)	(2.44 2.06 1.34)	2.00
AM_441_	(5.4 6.8 8.2)	(0.46 0.32 0.18)	(2.48 2.18 1.48)	2.11
AM_442_	(3.4 5.3 7.2)	(0.38 0.26 0.14)	(1.29 1.38 1.01)	1.30
AM_443_	(4.2 5.9 7.6)	(0.5 0.35 0.2)	(2.1 2.06 1.52)	1.98

^a^Weak attributes

**Table 11:** Weaker attributes based on the current state and recommended suggestions

Weak attribute	Suggestions for improvement
Transparent information flow *(AM*_*113*_ *)*	• Conduct group meeting • Circulate minutes and review on caregivers feedback • Discuss the detailed dispensary plan/roadmap to all caregivers
Adequate salary and bonuses for caregivers *(AM*_*134*_ *)*	• Link the incentives system to innovative ideas/improvements •Introduce bonus payment for duty on festival sessions • Attractive salary packages
Avoid the medical descriptions reading error *(AM*_*212*_ *)*	• Medical descriptions are printed through computer systems
In house/nearby pathology laboratory services (AM_223_)	• Setup the pathology laboratory in the premises of dispensary area
Technical up-gradation of dispensary equipments and facilities *(AM*_*232*_ *)*	• Modernise the medical equipments • For saving time, use new technology and methods
Minimization of patient throughput time *(AM*_*243*_ *)*	• Analyse the patients arrival pattern and allocate more duty doctors in peak hours
Adequate training programme for safety practices *(AM*_*431*_ *)*	• Conduct frequent training program for safety practices to caregivers • Provide adequate hands-on training to caregivers for fire fighting • Fix smoke detectors and automatic water sprinkler systems inside the dispensary

### Statistical validation of fuzzy results

Applicability of fuzzy logic approach in assessment of healthcare agility has been statistically validated following the literature [28]. To accomplish this step, a feedback session was conducted with 5 caregivers and 5 patients of the case dispensary. Among the care givers, 3 were doctors and 2 were senior nurses on duty at the dispensary. Similarly the 5 patients chosen for the feedback session were the patients who visited the dispensary frequently in last one year. The above respondents were selected randomly from the pool of caregivers and patients. The decision for going with 5 caregivers and 5 patients was based on literature [29]. The respondents were asked to rate the agile criteria of the dispensary in a Likert’s scale ranging from 0–10. The maximum and minimum mean rating was found to be 5.8 and 7.8 respectively which confirmed the FAMI range obtained in fuzzy calculation. Table 12 shows the rating of the respondents and the mean scores. Following this, a *t*-test was performed on the mean rating to examine whether the ‘assessment of agility using fuzzy logic is accepted or not’. At first, the test value was assigned as ‘10’ which indicates that the null Hypothesis (H_0_) is “H_0_: 100% of the feedback opinions favoured the assessment results of agility” at 95% confidence interval”. The significance of two tailed *t*-test (*p*-value) was found to be less than 0.05 and as a result, the null hypothesis was rejected at 95% level of confidence. Then, the test value was lowered to 9, 8 and 7 in subsequent attempts and the significance (*p*-value) converged towards 0.05 between 7.1 and 7.19. This indicated that the null hypothesis can’t be rejected for test value between 7.1 and 7.19. Statistically, this result translated into “71% of the feedback opinions favoured the assessment results of agility” at 95% confidence interval. In summary, it was established that assessment of agility using fuzzy logic can be adopted with a success rate of 71%.

**Table 12:** Mean responses of the caregivers and patients

S.No	Agility Criteria	Rating in Likert’s scale of range 0-10										Mean Response
		D1	D2	D3	D4	D5	D6	D7	D8	D9	D10	
1	Management structure of dispensary	5	6	6	6	5	7	7	7	8	5	6.2
2	Autonomy of work force	7	6	7	6	6	7	6	7	6	7	6.5
3	Top management support	9	7	6	6	7	6	6	6	7	6	6.6
4	IT integration	7	6	7	6	5	7	6	6	5	7	6.2
5	Automation of equipments	7	6	6	6	5	6	5	6	4	7	5.8
6	Change in service and technical process	6	6	6	7	6	6	7	7	6	6	6.3
7	Patient flow and time management	6	7	8	8	7	9	6	9	7	6	7.3
8	Visual Control	5	6	7	6	7	5	6	7	7	6	6.2
9	Patients’ response adaptation	5	6	7	7	6	7	7	6	6	9	6.6
10	Status of the Caregivers	7	6	7	6	6	7	6	6	6	7	6.4
11	Flexible work practices	6	7	6	8	6	6	8	6	8	9	7
12	Patients’ service quality	9	7	9	7	8	9	7	6	7	9	7.8
13	Optimum treatment cost	7	6	5	6	5	5	6	7	6	7	6
14	Patient and caregivers safety	9	7	9	8	9	6	7	7	7	8	7.7
15	Caregivers involvement	9	8	7	8	7	6	6	6	5	6	6.8
Mean		6.9	6.5	6.9	6.7	6.3	6.6	6.4	6.6	6.3	7.0	

## Results and discussions

The case analysis brings forth two essential insights. First, the current agility level of the dispensary is “Agile” which is average in relation to the Agility Labels (AL). Second seven attributes viz. transparent information flow, adequate salary and bonuses for caregivers, medical descriptions reading error, in house/nearby pathology laboratory services, technical up-gradation of dispensary equipments and facilities, minimization of patient throughput time and adequate training programme for safety practices need the attention of the management to enhance the dispensary’s agility. Though, this study deals with only one healthcare organization, the means of assessment and the findings regarding the critical attributes can be extended to other healthcare organizations. Moreover, it delineates the framework for healthcare agility assessment which would help both management and researchers in the field to extend and enrich it further.

In the context of university dispensary, the study recommends that the managers should conduct group meetings, circulate the minutes, review the caregiver’s feedback and discuss the management plan with all caregivers. This kind of communication would help the frontline employees to remain updated with information and plan the workflows accordingly. In addition, the management should link the incentive system to innovative and creative performances of the employees and introduce bonus for the staff on duty in festive occasions. This step would motivate the employees to be proactive and flexible while discharging the duty. Apart from this, “error in reading the medical description by the pharmacists/nurses” and “absence of in house pathology laboratory service” were found to be another set of challenges before dispensary agility. The management can address these issues by providing printed medical descriptions instead of handwritten ones and setting up a pathology laboratory inside the dispensary premises. In addition, technical up-gradation of the equipments and reduction of patient throughput time were also found to be crucial for the agile capability of the dispensary. The management should upgrade the medical equipments and allocate more doctors in peak hours. Apart from this, frequent training programs on fire safety practices and installation of smoke detectors and automatic water sprinklers would help enhance the agile performance of the dispensary. The suggestive measures recommended to the management are presented in Table 11.

## Conclusions

In conclusion, this study suggests that agile concepts are beneficial for healthcare organizations both in terms of meeting the service demand which changes quickly and unpredictably and retaining its competitive advantage over other players in the field. In this context, assessment of agility plays a crucial role because it helps to understand how flexible, accommodative and responsive is the healthcare organization at present and what prevents it from being more flexible and accommodative. According to our findings transparent information flow, adequate salary and bonuses for caregivers, elimination of medical descriptions reading error, in house pathology laboratory services, technical up-gradation of medical equipments, minimization of patient throughput time and adequate training programme for safety practices are imperative for healthcare agility. However, generalizability of these findings can be obtained by replicating the study in other healthcare organizations. Therefore, we propose that future research should replicate the study in large multi-speciality hospitals in different geographical locations. Apart from this, we suggest future research to explore the cause and effect paradigm for each of the attributes discussed in the study. This would help extend the current study and enrich the body of knowledge with new insights.
